# Can the two mechanisms of tumor cell killing by radiation be exploited for therapeutic gain?

**DOI:** 10.1093/jrr/rrt111

**Published:** 2013-10-08

**Authors:** J.D. Chapman

**Affiliations:** CRM Consulting Services, West Kelowna, BC, V4T 3H7, 2167 Madera Court, Canada

**Keywords:** LQ model, tumor cell killing, TCP modeling, NTCP modeling, optimal radiotherapy

## Abstract

The radiation killing of tumor cells by ionizing radiation is best described by the linear–quadratic (LQ) model. Research into the underlying mechanisms of α- and β-inactivation has suggested that different molecular targets (DNA in different forms) and different microdosimetric energy deposits (spurs versus electron track-ends) are involved. Clinical protocols with fractionated doses of about 2.0 Gy/day were defined empirically, and we now know that they produce cancer cures mainly by the α-inactivation mechanism. Radiobiology studies indicate that α and β mechanisms exhibit widely different characteristics that should be addressed upfront as clinical fractionation schemes are altered. As radiation treatments attempt to exploit the advantages of larger dose fractions over shorter treatment times, the LQ model can be used to predict *iso*-effective tumor cell killing and possibly *iso*-effective normal tissue complications. Linking best estimates of radiobiology and tumor biology parameters with tumor control probability (TCP) and normal tissue complication probability (NTCP) models will enable us to improve and optimize cancer treatment protocols, delivering no more fractions than are strictly necessary for a high therapeutic ratio.

## INTRODUCTION

A linear–quadratic (LQ) relationship precisely describes the radiation killing of human tumor cells when important experimental factors are controlled [1]. The most important are the use of cell populations of homogeneous radiosensitivity and the minimization of sublesion damage repair during radiation exposures. The former is best achieved with synchronized cells and latter with high dose-rates or low temperatures that inhibit repair enzyme function. When these conditions are met, cell killing is well described by the product of two Poisson escape probabilities for single-hit and double-hit inactivation events, respectively:
(1)
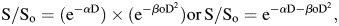

where, S/S_o_ = surviving fraction, α = the single-hit inactivation coefficient, β_o_ = the maximal double-hit inactivation coefficient (no repair) and D = dose.

This equation has been used to describe radiobiology data by a number of investigators for many years (see Lea [2]) but in the 1970s was related to important physical (microdosimetry) and biophysical (DNA structure) considerations by Kellerer and Rossi [3] and Chadwick and Leenhouts [4], respectively. To avoid misunderstanding, we recommend that α and β_o_ be used only to describe the inactivation rates of homogeneous cell populations irradiated in this manner [5].

### Biophysical implications of the LQ model

In the 1970s, my laboratory adopted the procedure of irradiating mammalian cells at 4–6°C so that maximal values of βo would be expressed in their cell survival curves. The radiation sources available for these experiments produced dose-rates of 1–2 Gy/min at positions convenient for cell exposures. And most studies were performed with G_1_-phase, G_o_-phase or mitotic cells so that inactivation parameters would be expressed per diploid or tetraploid genome. Those studies confirmed that:

- repair of α-inactivating events never occurred but that the molecular damages which lead to β-inactivation could be completely repaired [6],
- the indirect action of OH^**·**^ accounted for about 85% and 50% of aerobic cell killing by the α- and √β-inactivation mechanisms, respectively [7],
- the oxygen enhancement ratio for α-inactivation was lower (1.8–2.0) than that for √β-inactivation (∼3.0) [8, 9], and
- DNA in compacted form correlated with extreme sensitivity by α-inactivation [6, 10]. In fact, cells at mitosis (where DNA is maximally compacted) expressed radiation sensitivities similar to those of several repair-deficient cell lines [9].

Monte Carlo simulations of electron stoppage in water indicated that track-ends produced larger volumes of specific energy depositions than did the average 60 eV events radiation chemists have named ‘spurs’ [11, 12]. The vast majority of the absorbed dose delivered by photon and electron beams in tumor cells will be spur-like (diffusion and reaction distances of about 5 nm), while a much smaller portion will be deposited in 700 eV track-end events over larger volumes (reaction distances of about 15–20 nm) [1]. Consequently, it was postulated that α-inactivation resulted from DNA lesions produced by ionizations within electron track-ends (ETELs) that superimposed a radiosensitive component of compacted chromatin [11], while β-inactivation resulted from simple DNA damage such as base alterations, single-strand breaks and double-strand breaks produced mainly by spurs [1]. Since the molecules of living cells are well hydrated, water radicals would be important for both mechanisms. Other studies of DNA damage and charged particle effects on cells identified similar lesions responsible for α-inactivation and described them as multiple lethally damaged sites (MLDSs) and clustered lesions (CLs), respectively [13, 14]. This research provided a plausible molecular framework upon which investigation of tumor cell killing could be based and presented multiple avenues for further biophysical investigation. The proportion of cell killing by these two mechanisms is dependent upon dose fraction size, and current practice (∼2 Gy/day) has empirically optimized tumor cell killing by the α-mechanism. The ‘famous’ α/β ratio is the dose at which these mechanisms contribute equally to total cell killing (we currently teach that this is ∼ 10 for tumor cells). As hypofractionation techniques are clinically implemented, the proportion of tumor cell killing by the β-mechanism will increase and its unique properties will become more important for understanding tumor responses.

The majority of radiobiology research does not meet these rigorous conditions for experimentation, but the LQ equation continues to be used to describe a variety of different experimental results, including patient response. Nevertheless, the LQ models developed by microdosimetric [3] or by biophysical [4] considerations require that these conditions be met if molecular understanding of radiation effects is to be gained. The most important factors in this regard, the homogeneity of the radiosensitivity of cells under investigation and the dose-rate and temperature during the radiation exposures, should be essential information for any study presented for publication.

### What about cell populations of mixed radiosensitivity?

The LQ equation has often been used to describe the radiation inactivation of mixtures of cells of different intrinsic radiosensitivity. For example, when surviving fractions of asynchronous populations were best-fitted by this equation, the resultant α and β parameters were some combination of the parameters which describe the various subsets [15]. We recommend that such parameters be indicated with an over-bar to distinguish them from the parameters derived from cell populations of homogeneous radiosensitivity.
(2)
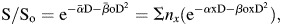

where, *n*_*x*_ is the proportion of the cell population with inactivation parameters, α_x_ and β_ox_.

Table 1 shows ᾱ and √β̄_0_ values obtained for ten different human tumor cell lines (growing asynchronously) irradiated at low temperature to inhibit sublesion damage repair. It is apparent that the variation in ᾱ-inactivation parameters of these cells is large (60-fold), whereas the variation in √β̄-inactivation parameters is quite small (±27%). Studies with synchronized populations of Chinese hamster lung fibroblasts and with human tumor cell lines indicated that α-inactivation was relatively constant throughout the interphase of the cell cycle [15, 16] when the data were corrected for genome multiplicity. These interphase values of α correlated with the order of radiosensitivity exhibited by asynchronous populations at low dose. Consequently, it is reasonable to assume that the intrinsic radiosensitivities of human tumor cell lines and tumor cells released from biopsy specimens will predict for the radioresponsiveness of like tumors *in vivo*.

**Table 1.** Parameters of intrinsic *in vitro* radiosensitivity of various asynchronous populations of human tumor cell lines

Cell line	Tumor origin	ᾱ(Gy^−1^)	√β̄(Gy^−1^)	SF_2Gy_
HT-29	colon	0.03	0.25	0.73
TSU	prostate	0.06	0.22	0.70
OVCAR10	ovary	0.16	0.24	0.58
PC-3	prostate	0.24	0.26	0.48
DU-145	prostate	0.31	0.22	0.48
MCF-7	breast	0.38	0.16	0.43
A2780	ovary	0.47	0.27	0.29
LnCap	prostate	0.49	0.12	0.25
HT144	melanoma	1.43	0.36	0.03
Mo59J	glioblastoma	1.80	0.31	0.01

Average √β̄ = 0.241 ± 0.065.

The bulk of information in the literature on intrinsic tumor cell radiosensitivity is reported as surviving fraction after a radiation dose of 2 Gy (SF_2Gy_). If we assume that the √β̄_o_ parameter for human tumor cells is relatively invariant and equal to 0.241 Gy^−^^1^ (the value shown in Table 1), a distribution of α parameters can be computed from the SF_2Gy_ for the different classes of human tumor cell lines reported by Deacon *et al*. [17] using the equation:
(3)
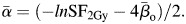

This assumption was deemed warranted, since the β mechanism contributes only a small proportion of cell killing at 2 Gy for the majority of human tumor cells and exhibited little variation. Table 2 gives the values of ᾱ-inactivation for the 51 cell lines reported [17], along with estimates of error (SD). These have been grouped into only three classes since Groups A and B and Groups C and D had similar radiosensitivities. We regard these values to be reasonable and representative for input into tumor control probability (TCP) models of tumor response. Note that the α/β ratios for these groups of tumor cell lines are 12.6, 6.2 and 4.5, a refinement of the standard value of ∼10 that we teach to medical residents. When similar analyses are performed on the SF_2Gy_ data reported by West *et al*. [18] and Björk-Eriksson *et al.* [19] for tumor clonogens released from cervical and head and neck cancers, respectively, average values of ᾱ ± SD were obtained (also shown in Table 2). These agree well with the values obtained for the histologically similar cell lines grouped as C and D. This analysis gives us confidence that the intrinsic radiosensitivities of human tumor cells lines will reflect those of clonogenic cells derived from biopsies of tumors of similar pathology. Table 2 also shows the average values of ᾱ and √β̄_o_ reported in the literature for human prostate tumor cell lines [20, 21]. It is likely that this value of √β̄_o_ is lower than 0.241 Gy^−^^1^ (the average in Table 1) since most of the studies were performed at room temperature or at 37°C, where some repair of the sublesions of β-killing would be expected during radiation exposures.

**Table 2.** Intrinsic radiosensitivity parameters for various human tumor cell lines and for cells released from tumor biopsies

Tumor histology	ᾱ-parameter	√β̄-parameter
**Groups A and B**: comprising lymphoma, myeloma, neuroblastoma, medulloblastoma and SSLC	0.73 ± 0.23 Gy^−1^	0.241 Gy^−1^
**Groups C and D**: comprising breast, bladder, cervical carcinoma, pancreatic, colorectal and squamous lung cancer	0.36 ± 0.25 Gy^−1^	0.241 Gy^−1^
**Group E**: comprising melanoma, osteosarcoma, glioblastoma renal carcinoma and	0.26 ± 0.17 Gy^−1^	0.241 Gy^−1^
Cervical carcinoma [17]	0.35 ± 0.21 Gy^−1^	0.241 Gy^−1^
Head and neck carcinoma [18]	0.40 ± 0.21 Gy^−1^	0.241 Gy^−1^
Prostate carcinoma [20]	0.26 ± 0.17 Gy^−1^	0.177 Gy^−1^

### Sublesion repair during radiation exposures

When laboratory studies of cell radiosensitivity are performed with dose-rates of 1–2 Gy/min and at temperatures of 22–37°C, the repair of sublesions of the β-mechanism must be accounted for. Our research indicated that a first-order repair coefficient (DNA strand break repair) predicted well for the reduced cell killing observed under such conditions [12]. Several survival curves generated with Chinese hamster fibroblasts at various temperatures and dose-rates were best-fit to the following equation and yielded a sublesion repair rate of 0.03/min at 37°C:
(4)
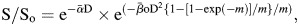

where ᾱ and β̄_o_ were obtained from studies performed at ∼4°C, *m* = *kD*/*R*, *k* = repair rate of β-mechanism sublesions at the specific temperature of interest, *D* = radiation dose and *R* = dose-rate.

It will be important to confirm that this sublesion repair rate is similar to that of human tumor cells so that realistic estimates of radiation cell killing can be obtained for *in vivo* exposures. DNA repair research also indicated that a small proportion of strand breaks might require a different enzyme system that requires longer times [22]. In most cases, our radiobiology studies were not precise enough to address this second component of repair, although it could be important in patients for minimizing normal tissue complications.

### Tumor cell killing by fractionated treatment protocols

When radiation is administered in daily fractions of ∼2Gy to cancer patients, the total tumor cell killing over the weeks of treatment can control cancer growth and produce some cures. The tumor tissue response during these treatments has been explained by the 4 or 5 Rs of radiobiology [23, 24]. To be effective, the dose fraction size must be large enough to eradicate a number of tumor cells each day that is greater than any increase due to tumor growth (repopulation). The selective killing of the most radiosensitive tumor cells in the cell cycle will leave behind a more resistant population that can redistribute to more radiosensitive phases prior to subsequent doses. The selective killing of oxygenated cells will, over time, make available an oxygen supply to those cells that were hypoxic and, consequently, radioresistant. Repair of sublethal damage between fractions will result in less tumor cell kill but will have the major advantage of restoring normal tissues. And the intrinsic radiosensitivity of the tumor clonogens (mainly ᾱ-parameter) will be a major determinant of ultimate tumor response. Table 2 gives average ᾱ-parameters of various human tumor phenotypes that correlate with their radioresponsiveness *in vivo* and are appropriate for input into TCP models.

When fractionated doses are administered to human tumor cells *in vitro* or *in vivo*, cell killing will be expressed by the following equation:
(5)
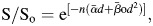

where *n* = fractions of dose *d* to deliver a total dose of *nd*.

**Fig. 1.**
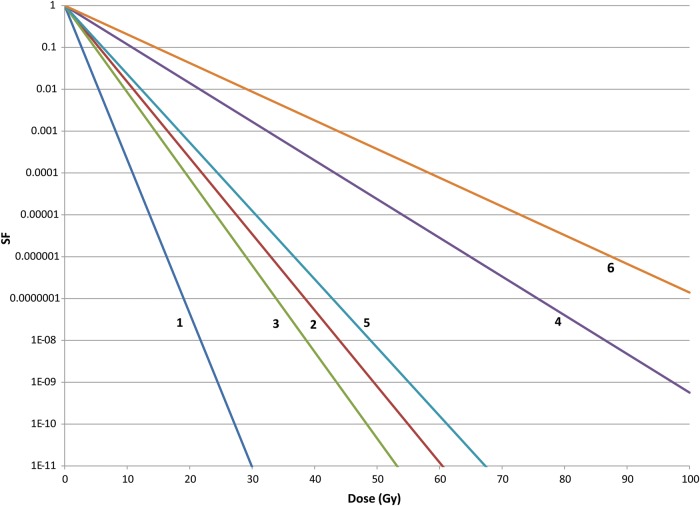
The surviving fraction of aerobic and hypoxic tumor clonogens irradiated with 2 Gy fractions whose α and √β inactivation parameters are those of Groups A and B (lines 1 and 2), Groups C and D (lines 3 and 4) and Group E (lines 5 and 6) tumors of Table 2.

Figure 1 shows the cell killing expected for aerobic and hypoxic tumor cells with intrinsic radiosensitivities of Group A and B, Group C and D and Group E of Table 2 when treated with 2 Gy dose fractions. The oxygen enhancement rations for α and √β parameters were 1.8 and 3.0, respectively [8, 25]. These curves assume no cell proliferation between the fractions, but this can be incorporated into the model, if known. Survival curves can be generated with each tumor type in response to dose fractions of any size and iso-effectiveness (equal cell killing) will be apparent.

### Clonogen density in solid human tumors

A most important parameter for determining the therapeutically effective dose is the number of tumor cells (clonogens) that must be killed to produce tumor cures. Unfortunately, reliable predictive assays for determining this and other important tumor properties important for prescribing optimal radiation treatments for individual patients have not been devised [26]. Assays using the Cell Adhesive Matrix (CAM) assay [27] or the soft agar assay [28] for determining cell radiosensitivity have severe limitations and have not been reproducible in different laboratories. Thus we rely on the data from human tumor cell lines of different phenotypes in the current literature (see Table 2). With regard to tumor clonogen number, the studies of West *et al*. [18] and Björk-Eriksson *et al*. [19] show that only 0.1–1.0% of all viable cells released by enzyme procedures from primary tumor biopsies produced colonies *in vitro*. If these cells are the ones that require inactivation for tumor cures, they constitute a small minority of the tumor mass. Other research that was directed to cancer drug development measured the solid tumor clonogen density to be ∼0.5 × 10^6^/g [29]. A value of clonogen density of 0.5–1.0 × 10^6^/g is consistent with these investigations. Since radiotherapy often treats planned tumor volumes (PTVs) of 0.01–1.01, the clonogen number that must be eradicated could be of the order of 10^7^–10^9^ or more.

### Transforming tumor cell killing into tumor cure probability

The LQ model can be used to generate tumor cure probability curves that reflect the various tumor parameters that are available. The mean number of surviving clonogens is given by:
(6)
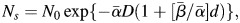

where, *N*_*s*_ = number of surviving clonogens, *N*_*0*_ = number of tumor clonogens at the start of treatment, ᾱ and β̄ are the best estimates of clonogen intrinsic radiosenstivity, *D* = total dose and *d* = fraction size. Equation 6 is simply a different form of Equation 5 and when the average values of ᾱ and √β̄ in Table 2 are used for cell killing by 2 Gy dose fractions with complete repair between the fractions, the curves in Fig. 1 are obtained. For tumors in Groups A and B that contain 10^8^ initial clonogens, eight logs of cell killing can be accomplished with less than 50 Gy total dose for either aerobic or hypoxic cells. For tumors in Groups C and D that contain the same number of initial clonogens, eight logs of cell killing can be achieved with < 50 Gy for aerobic cells but not for hypoxic cells. And for tumors in Group E (the most radioresistant class) that contain 10^8^ initial clonogens, eight logs of cell killing of aerobic cells is just possible with 50 Gy total dose, but more than double that dose will be required to kill a similar number of hypoxic clonogens. Consequently, tumor hypoxia will be an important factor for the killing of clonogens in tumors of class C and D and class E. Unfortunately, hypoxia is another tumor property that is not determined on a regular basis today.

**Fig. 2.**
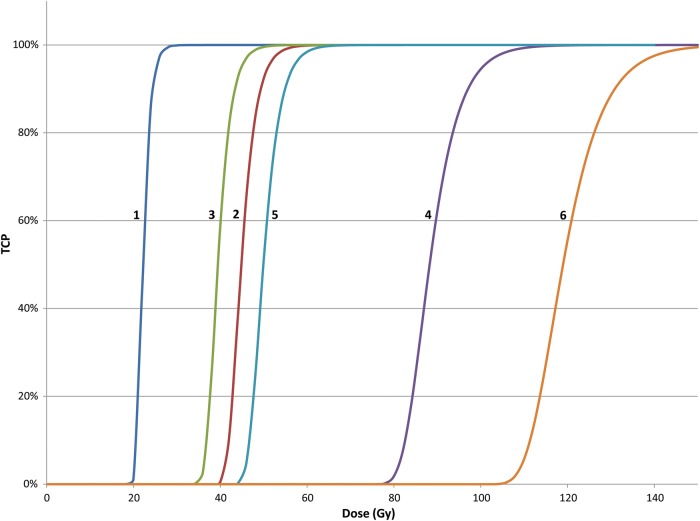
The tumor control probability (TCP) for aerobic and hypoxic tumors with radiosensitivities of Groups A and B, Groups C and D and Group E tumors that require the inactivation of 10^8^ clonogens for cure.

The Poisson probability of there being zero surviving clonogens at the end of a fractionated treatment is given by:
(7)
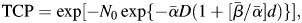

where, TCP = tumor control probability and the other parameters are as defined as in Equation 6 [30]. Equation 7 applies to tumor populations whose clonogens have identical ᾱ and √β̄ parameters and where there is no cell proliferation between the dose fractions. Figure 2 shows TCP values for patient populations with 10^8^ aerobic and hypoxic tumor clonogens whose intrinsic radiosensitivities are those of Groups A and B, Groups C and D, and Group E of Table 2. Again it is clear that tumors of Groups A and B with both aerobic and hypoxic clonogens should be cured with total doses of 50 Gy or less. For tumors of Groups C and D, those with aerobic clonogens should be treated well with 50 Gy but those with hypoxic cells will require much higher doses. And while most tumors of Group E with aerobic clonogens will be cured with doses of 60 Gy, those with hypoxic clonogens will require 110 Gy or more.

For those that attempt to fit the LQ model to clinical data (that will be even more heterogeneous), it is apparent that the slopes of human TCP curves are much more shallow than those shown in Fig. 2. Hypoxia, as discussed above, is one source of tumor response heterogeneity that can lead to the observed shallow response curves. How hypoxia should be factored into TCP modeling will only be known when such information is available for the various classes of tumors under treatment. For prostate cancer patients presenting for brachytherapy at the Fox Chase Cancer Center, measurements of tumor oxygenation were made with the Eppendorf electrode device [31]. Since tumors were extremely hypoxic or well oxygenated, tumor response was modeled with an increasing proportion of patients with hypoxic (radioresistant) tumors as tumor grade increased according to PSA expression [32]. Reasonable fits to clinical data for both brachytherapy and fractionated conformal therapy were obtained when reasonable estimates of clonogen numbers were used [21]. That study also used the average values of ᾱ and β̄ for prostate cancer cells lines reported in the literature (those shown in Table 2). No attempt was made to model possible tumor reoxygenation since measurements of tumor oxygenation were not made during the treatments. When tumor response modeling relies on fundamental parameters which are best estimates or even guesses, numerous outcomes are possible which might have little or no benefit for the clinic.

Another basis for the shallower slopes of tumor response for clinical outcome data was recognized to be related to an inherent distribution of ᾱ parameters for the clonogens in tumors of the same class in different patients. This is apparent from the cervix, head and neck and prostate data shown in Table 2. When a Gaussian distribution is included in Equation 7 to account for the heterogeneity of ᾱ-coefficients of the clonogens in tumors of the same histological class, a shallower TCP curve is obtained [33]. TCP modeling that does not take into account the expected variation in radiation inactivation parameters between like tumors in different patients should not be expected to yield accurate results.

A third basis for intratumor heterogeneity of intrinsic radiosensitivity parameters is the proportion of clonogens that are quiescent at the time of irradiation. Our research showed that cells in G_0_-phase expressed α-inactivation values about one-half that of diploid cells undergoing cell division (G_1_-phase cells) [12]. When mammalian cells are irradiated in multicellular spheroids, they are typically more resistant to radiation killing [34, 35]. Consequently, the distribution of α-inactivation parameters in the clonogens of human tumors might be broader due to their growth status. It is assumed that slow-growing tumors will have a smaller number of clonogens undergoing active proliferation than the more rapidly growing tumors. Again, the approximate growth rates of individual tumors in patients is not usually determined prior to treatment prescription, since getting on with tumor cell killing is a priority for successful outcomes.

Will there come a day when prescriptions of radiation treatment for individual patients can be customized to the radiation and tumor biology parameters of the clonogens in their cancers? The answer is ‘maybe’. While predictive assay research was a noble venture in the 1980s [26], the definition of practical and reliable methods of determining the intrinsic radiosensivity, the number, the quiescent/proliferation fraction of clonogens and the hypoxic fraction of clonogens in individual human tumors was never realized. Without such data, TCP modeling will be restricted to patient populations with like tumors whose interpatient variations of clonogenic parameters will blur the data outcome. This does not mean that TCP modeling will have little or no value for predicting the results of novel clinical strategies, especially those of altered dose fractionation, but that research will have to be performed with groups of patients. This is the basis for current research by RTOG and ESTRO and will pose no serious problem for implementing different treatment strategies with potential benefit.

In that regard, the move to hypofractionated treatments for most cancers should be implemented cautiously with adequate time for follow-up evaluation. Since PTVs can be defined with improved accuracy, and the delivery of dose to those PTVs can be much more conformal with our modern technologies, higher dose fractions (3–5 Gy) on 2, 3 or 5 days a week should be investigated for improved tumor response and lower normal tissue complications. Radiation oncology research should determine whether current tumor response rates and cures might be achieved with significantly fewer patient setups and shorter overall times with no increase in normal tissue complications. Significant cost savings to health care systems and less stress on cancer patients would be a noble goal of such clinical research. Again, a move in this direction should be based upon the wealth of tumor biology research performed over the past 40 years that can inform about expected results.

With regard to NTCP modeling [36], the fundamental mechanisms of early and late normal tissue complication induction by radiation are even less well defined. In those cases where normal tissue stem cell killing produces a detrimental effect, the LQ model could play a role. In fact several radiobiology studies have been performed with fibroblasts from various normal tissues. But when interactions between stromal and vascular elements produce deleterious effects, our knowledge of underlying mechanisms is limited. Thus the modeling of normal tissue complications after different radiation treatments will be less quantitative even when the LQ model is employed [37]. When fractions of higher dose are employed, tumor response may result from mechanisms other than tumor cell killing (eg. stromal and vasculature effects) for which the LQ model might not obtain [38].

It is my opinion that our current understanding of the two mechanisms of tumor clonogen killing by ionizing radiation can be exploited to define improved dose fractionation schemes that should make radiation oncology less costly and a less stressful treatment for cancer patients in the upcoming years. Laboratory and clinical studies are urgently needed to better define the tumor biology parameters important for input into TCP and NTCP models. What is not helpful at this time are more mathematical ‘gymnastics’ based upon imprecise tumor biology parameters.

## References

[1] Chapman JD (2003). Single-hit mechanism of cell killing by radiation. Int J Radiat Biol.

[2] Lea DE (1962). Cambridge: The University Press. Action of Radiations on Living Cells.

[3] Kellerer AM, Rossi H (1972). The theory of dual radiation action. Top Radiat Res Q.

[4] Chadwick KH, Leenhouts HP (1973). The molecular theory of cell survival. Phys Med Biol.

[5] Chapman JD, Gillespie CJ (2012). The power of radiation biophysics – let's use it. Int J Radiat Oncol Biol Phys.

[6] Chapman JD, Stobbe CC, Gales T (1999). Condensed chromatin and cell inactivation by single-hit kinetics. Radiat Res.

[7] Chapman JD, Doern SD, Reuvers AP (1979). Radioprotection by DMSO of mammalian cells exposed to X-rays and to heavy charged-particle beams. Radiat Environ Biophys.

[8] Chapman JD, Gillespie CJ, Reuvers AP (1975). The inactivation of Chinese hamster cells by X-rays: the effects of chemical modifiers on single- and double-hit events. Radiat Res.

[9] Stobbe CC, Park SJ, Chapman JD (2003). The radiation hypersensitivity of cells at mitosis. Int J Radiat Biol.

[10] Chapman JD, Stobbe CC, Matsumoto Y (2001). Chromatin compaction and tumor cell radiosensitivity at 2 Gy. Am J Clin Oncol.

[11] Chapman JD, Meyn RE, Withers HR (1980). Biophysical models of mammalian cell inactivation by radiation. Radiation Biology in Cancer Research.

[12] Chapman JD, Gillespie CJ (1981). Radiation-induced events and their time-scale in mammalian cells. Adv Radiat Biol.

[13] Ward JF (1982). Some biochemical consequences of the spatial distribution of ionizing radiation-produced free radicals. Radiat Res.

[14] Goodhead DT (1994). Initial events in the cellular effects of ionizing radiations: clustered damage in DNA. Int J Radiat Biol.

[15] Gillespie CJ, Chapman JD, Reuvers AP (1975). The inactivation of Chinese hamster cells by X-rays: synchronized and exponential cell populations. Radiat Res.

[16] Biade S, Stobbe CC, Chapman JD (1997). The intrinsic radiosensitivity of some human tumor cells throughout their cell cycles. Radiat Res.

[17] Deacon J, Peckham MJ, Steel GG (1984). The radioresponsiveness of human tumors and the initial slope of the cell survival curve. Radiother Oncol.

[18] West DML, Davidson SE, Roberts SA (1993). Intrinsic radiosensitivity and prediction of patient response to radiotherapy of carcinomas of the cervix. Brit J Cancer.

[19] Björk-Eriksson T, West CML, Karlsson (2000). Tumor radiosensitivity (SF_2Gy_) is a prognostic factor for local control in head and neck cancers. Int J Radiat Oncol Biol Phys.

[20] Algan O, Stobbe CC, Helt AM (1996). Radiation inactivation of human prostate cancer cells: role of apoptosis. Radiat Res.

[21] Nahum AE, Movsas B, Horwitz EM (2003). Incorporating measurements of hypoxia into tumor local control modeling of prostate cancer: implications for the α/β ratio. Int J Radiat Oncol Biol Phys.

[22] Dugle DL, Gillespie CJ, Chapman JD (1976). DNA strand breaks, repair and survival in X-irradiated mammalian cells. Proc Natl Acad Sci U S A.

[23] Withers HR (1975). The four Rs of radiotherapy. Adv Radiat Biol.

[24] Steel GG, Peacock JH (1989). Why are some tumors more radiosensitive than others?. Radiother Oncol.

[25] Palcic B, Skarsgard LD (1984). Reduced oxygen enhancement ratio at low doses of ionizing radiation. Radiat Res.

[26] Chapman JD, Peter LJ, Withers HR (1988). Prediction of Tumor Treatment Response.

[27] Baker FI, Spitzer G, Ajani JA (1986). Drug and radiation sensitivity measurements of successful primary monolayer culturing of human tumor cells using cell-adhesive matrix and supplemented media. Cancer Res.

[28] Courtney VD, Mills J (1978). An *in vitro* assay for human tumors grown in immune-suppressed mice and treated *in vivo* with cytotoxic agents. Brit J Cancer.

[29] Baker F, Sanger I (1991). The density of clonogenic cells in human solid tumors. Int J Cell Cloning.

[30] Nahum AE, Sanchez-Nieto B (2001). Tumor control probability modeling: basic principles and applications in treatment planning. Phys Med.

[31] Movsas B, Chapman JD, Hanlon AL (2002). A hypoxic ratio of prostate pO_2_/muscle pO_2_ predicts for biochemical failure in prostate cancer patients. Urology.

[32] Movsas B, Chapman JD, Greenberg RE (2000). Increasing levels of hypoxia in human prostate carcinoma correlate significantly with increasing clinical stage and age: an Eppendorf pO_2_ study. Cancer.

[33] Sanchez-Nieto, Nahum AE (1999). The Delta-TCP concept: a clinically useful measure of tumor control probability. Int J Radiat Oncol Biol Phys.

[34] Durand RE, Sutherland RM (1972). Effects of intercellular contact on repair of radiation damage. Exp Cell Res.

[35] Durand RE, Sutherland RM (1973). Growth and radiation survival characteristics of V79-171b Chinese hamster cells: a possible influence of intercellular contact. Radiat Res.

[36] Burman C, Kutcher GJ, Emami B (1991). Fitting of normal tissue tolerance data to an analytic function. Int J Radiat Oncol Biol Phys.

[37] Marks LB, Ten Haken RK, Martel MK (2010). Guest editor's introduction to QUANTEC: a user's guide. Int J Radiat Oncol Biol Phys.

[38] Song CW, Cho LC, Yaun J (2013). Radiobiology of stereotactic body radiation therapy/stereotactic radiosurgery and the linear-quadratic model. Int J Radiat Oncol Biol Phys.

